# Factors Influencing Women's Preferences for Places to Give Birth in Addis Ababa, Ethiopia

**DOI:** 10.1155/2015/439748

**Published:** 2015-08-30

**Authors:** Yibeltal Tebekaw, Yohana James Mashalla, Gloria Thupayagale-Tshweneagae

**Affiliations:** Department of Health Studies, University of South Africa, Pretoria, South Africa

## Abstract

The main aim of this study was to examine factors determining women's preference for places to give birth in Addis Ababa, Ethiopia. A quantitative and cross-sectional community based study design was employed. Data was collected using structured questionnaire administered to 901 women aged 15–49 years through a stratified two-stage cluster sampling technique. Multinomial logistic regression model was employed to identify predictors of delivery care. More than three-fourth of slum women gave birth at public healthcare facilities compared to slightly more than half of the nonslum residents. Education, wealth quintile, the age of respondent, number of children, pregnancy intention, and cohabitation showed net effect on women's preference for places to give birth. Despite the high number of ANC attendances, still many pregnant women especially among slum residents chose to deliver at home. Most respondents delivered in public healthcare institutions despite the general doubts about the quality of services in these institutions. Future studies should examine motivating factors for continued deliveries at home and whether there is real significant difference between the quality of maternal care service offered at public and private health facilities.

## 1. Introduction

Assurance of healthcare for all segments of the population with special attention given to the health needs of women and children was one of the top priorities in the Ethiopian Health Policy [1]. The endorsement of MDG 5 in the HSDPs is an indication of the commitment or political will of the government towards reducing maternal mortality across the nation [2]. Yet, Ethiopia's health system is underdeveloped and underfinanced [3]. While some progress has been made in providing basic health services to poor women and their children, the progress may be uneven because many people are not reached with services [4].

Ethiopia's total health expenditure as a percentage of the gross domestic product (GDP) has remained stable at 4.3% for years. With emphasis given to publicly funded healthcare, out-of-pocket payment constitutes 42% [5]. The public health sector is the main provider of primary healthcare and serves two-thirds of the population who cannot afford private healthcare [6]. The main objective of the public sector service provision, as stated in the National Health Policy, is “*to give comprehensive and integrated primary health care services in a decentralized and equitable fashion*” [1].

Childbirth and its process are one of the most significant life events to a woman [7]. The time of birth as well as shortly thereafter is the most dangerous period in a child's life especially in the developing world [7, 8]. Hence the choice of place of delivery for a pregnant woman is an important aspect of maternal healthcare. The place of delivery is an important factor often related to the quality of care received by the mother and infant for influencing maternal and child healthcare outcomes [7]. In Addis Ababa, the capital of Ethiopia, though the private health facilities (hospitals and clinics) outnumber public clinics [9], only 20% of deliveries take place in the private sectors and 17% of mothers deliver at home [4].

This study aims to systematically explore the differences and the factors that influence women's preferences for places to give birth in Addis Ababa. It is envisaged that a clear understanding of such factors is key in building a responsive maternal healthcare system and improving health outcomes in Ethiopia.

## 2. Research Design and Methods

### 2.1. Sampling and Data Collection

Addis Ababa, the study area, is divided into 10 subcities and each subcity is further divided into several small administrative units called Kebeles. In the 2007 Ethiopia Housing and Population Census, Kebeles were further subdivided into enumeration areas (EAs). An EA is a geographic area consisting of a convenient number of dwelling units which was used as a counting unit for the census. The average number of households (HHs) per EA in urban Ethiopia is 169. The number of clusters (EAs) in Addis Ababa was about 3865 [3].

Because of the different levels of political or administrative structures and wider geographic areas, cluster sampling technique was employed for this study. The study employed a stratified, two-stage cluster design. Since Addis Ababa is entirely urban, stratification was achieved by using the subcities (10 strata). Using the 2007 Population and Housing Census data, in the first stage 30 enumeration areas (EAs) were selected independently from all the strata with probability proportional to (EA) size (PPS) of households. In the second stage, 906 households were selected with PPS of households in each EA. The systematic random sampling of the eligible households was done based on the number of households recorded during the complete listing of households in each EA during the last EDHS 2011 [3]. To minimize sampling errors that may arise due to changes in the years after the last enumeration (complete household listing), approaching the subcity and Kebele administrations as well as community members in the respective EAs was an important step in the survey process. It was necessary to be aware of and be sensitive to the various community level dynamics in the study area. Hence, the demolition of significant portions of four EAs from four strata due to the city's reconstruction process was the major change reported and verified by the first author. Three EAs were replaced by other randomly selected three adjacent EAs and remaining households of the fourth EA were completed from a section of a randomly selected adjacent EA.

In this study verbal face-to-face interview was administered using a structured questionnaire. Recall bias was taken into consideration during the development of the questionnaire. Therefore, women were asked about their most recent or last birth and the date of birth of the child was asked. Ethical clearance was obtained from the National Research Ethics Review Committee of the Ministry of Science and Technology, Ethiopia. The target population was all women of 15–49 years of age who have experienced at least one birth in the last 1–3 years before the date of data collection, December 2013–January 2014.

### 2.2. Description of Variables

The independent variables for this study were selected based on a modified version of the Behavioural Model of Health Services [10]. The model distinguished three sets of factors related to healthcare seeking behaviour of individuals: the predisposing, enabling, and need factors.

Under the* predisposing factors*, demographic variables such as age, number of living children, current marital status, and pregnancy intention related to last childbirth and social structure variables such as education, occupation, and ethnicity were considered at individual, household, and community levels. As regards pregnancy intention, women were asked about their recent births whether they wanted it then, wanted it later, or did not want to have any more children at all. In the analyses, the intention status of the birth was further defined as a dichotomy variable: intended for births wanted by then versus unintended for being either mistimed or unwanted by then. Women's education was defined here as the highest level of schooling attended regardless of whether the woman completed the level.

Under the* enabling factors*, individual and family resource indicator variables including housing tenure, health insurance, and wealth quintile were included. Those who visited health facility for ANC were also asked whether there was an organization or agency that either partially or fully covered their expenses and the responses were grouped as “yes” or “no.” Housing tenure was categorised as the house in which the respondent lives is either owned by her or not owned by her, that is, owned versus rental. The type of residence was categorised as slum and nonslum residences based on the five indicators including access to improved water, access to improved sanitation, sufficient living area, durability of housing, and secure tenure (housing tenure) developed by United Nations Human Settlements Programme [11].

The outcome variable was “*preference for places to give birth*” which represented three choices that a woman can make:* deliver at a public healthcare facility*,* deliver at private healthcare facility,* and* deliver at home with or without professional assistance*.

### 2.3. Data Analysis

Data was entered using the Census and Survey Processing System (CSPro) software and was analysed for both descriptive and inferential statistics using the Statistical Package for Social Sciences (SPSS) version 16.0 [12]. Bivariate (Chi-square) tests were also applied. Multinomial logistic regression model was fitted to investigate the potential factors influencing preferred places to give birth. The base category was delivery at public healthcare institute. For this study, *P* < 0.05 was considered statistically significant at 95% confidence interval.

## 3. Results

In this study, the data collection response rate was 99.4%, 901 out of 906. Women were asked about their place of ANC follow-up and places of delivery during their last birth. The inquiry was about where the majority of the ANC visits occurred as there could be possibility of shifting from one place of care to another during the same pregnancy period.

### 3.1. Disparities by Socioeconomic and Demographic Characteristics of Respondents

More than two-thirds (69.2%) and slightly less than a quarter (24.0%) of all the study participants gave birth at public and private healthcare facilities, respectively. About 6.8% of them delivered at home. Both slum and nonslum residents accessed ANC mainly at public healthcare facilities. More than three-fourths of slum resident women gave birth at public healthcare facilities compared to slightly more than half of the nonslum residents. Higher proportion of the nonslum residents (41.7%) gave birth at private facilities compared to only 15.3% of the slum residents.

Table 1 presents the results of the association between socioeconomic, demographic, and healthcare variables and women's preferences for places to give birth. Younger age, having fewer number of children, having unintended pregnancy, and cohabitation were associated with delivery at public healthcare facilities. The educational status of women associated significantly negatively with delivery at home but positively with delivery at private healthcare facilities. The employment status of women did not show statistically significant association with preference for place of delivery. Ethnically, delivery at private healthcare facilities was more likely among the Tigraways followed by the Amharas. Women having health insurance coverage were more likely to deliver at healthcare facilities than those without. Similarly, slum residents were more than twice likely to deliver at home compared to the nonslum residents.

About 6.3% of the ANC attendees finally delivered at home. Only 1.7% of the private facility ANC clients delivered at home compared to 6.7% of the public facility clients. In terms of shifting between types of facility, 30.0% of the private facility ANC clients delivered at public healthcare facilities while 22.6% of the public facility ANC clients delivered at private facilities.

The mother's knowledge about danger signs of pregnancy showed an effect on women's preferences for place of delivery. About 94.9% of those women counselled about the danger signs of pregnancy delivered at a health facility compared to 92.1% of those who were not counselled (*P* = 0.01). About 81.7% and 72.8% of clients of private and public healthcare facilities respectively were counselled on danger signs of pregnancy during antenatal follow-up.

The reasons behind women's preference for their places of delivery were further explored. Hence, preference for public healthcare facilities was attributed to short distance (72.7% versus 36.4%; *P* = 0.000), perceived low cost service (6.1% versus 2.4%; *P* = 0.037), and experienced low cost service (16.7% versus 4.3%; *P* = 0.000). On the other hand, preference for private healthcare facilities associated positively with short waiting time (19.1% versus 8.7%; *P* = 0.000), perceived good quality of service (15.2% versus 7.6%; *P* = 0.001), experienced good quality of service (43.1% versus 20.6%; *P* = 0.000), perceived good approach of service provider (10.0% versus 5.7%; *P* = 0.034), and experienced good approach of service provider (22.9% versus 10.5%; *P* = 0.000). There was no statistically significant influence of families, friends, or husbands on women's preference for places to give birth (*P* > 0.05).

### 3.2. Determinants of Preferences for Places to Give Birth

In Table 2, the results of the logit model show that younger women and those with 0–2 living children were less likely to deliver at home (OR = 0.90 and OR = 0.24) compared to older women and those with three or more living children, respectively. Women who had unintended pregnancy for their last birth and those with no formal education were 2.1 and 3.6 times more likely to deliver at home compared to those with intended pregnancy and those with secondary and above educational attainment level, respectively.

Contrary to home delivery preference, women with unintended pregnancy were less likely to deliver at private healthcare facilities. Conversely, women with intended pregnancy were 1.75 times more likely to deliver at private healthcare facilities compared to those with unintended pregnancies. Similarly, women with no formal education (OR = 0.18) or with primary education (OR = 0.33) were less likely to deliver at private healthcare facilities compared to those with secondary and above education.

The wealth class to which the household of the respondent belongs was a significant factor in predicting the preferred place of delivery. The lower the wealth quintile, the greater the likelihood of delivering at home. Women who belong to the middle class wealth quintile were 2.79 times more likely to deliver at home compared to the household with high class wealth quintile. It was also found that the odds of delivery at private healthcare facilities were low among women of low wealth quintile (OR = 0.56) compared to those in high wealth quintile.

## 4. Discussion

Our multinomial regression model shows that young women have lower probability of giving birth at home compared to older women. In Uganda [13] and in urban Kenya [14], young women were found to be better users of skilled professional assistance. The same is true in Nepal and India where institutional delivery was more common among young mothers compared to older ones [15, 16]. It has been argued that older women may consist of more traditional cohorts and may resist modern healthcare services [17].

We have also shown in this study that women with two or less living children were more likely to deliver at public healthcare facilities than at home compared to those with three or more living children. Our findings support results of a study from Entebbe, Uganda, which showed that primigravidae were more likely to attend government hospitals [18]. It is possible that women with three or more living children claim to be experienced and see no reason to deliver at health facilities. Alternatively, negative previous experiences may deter women from delivering at health facilities thereby exposing themselves to the complications of childbirth.

In relation to pregnancy intention we observed that women whose last pregnancy was unintended preferred delivery at home (OR = 2.11). Although the reasons for not utilising health facilities for unintended pregnancies are not very clear, it may be related to intention to conceal the delivery, lessened employment opportunities or lack of income, and delayed prenatal care [19–21].

Education is one of the key social determinants of health and healthcare. Low levels of female education [22] and lack of empowerment prevent women from seeking maternal care [23]. In the current study, women with no formal education were almost four times more likely to deliver at home compared to those with secondary and above educational level. If they use health facilities, those with no formal education and those with primary level of education were 82% and 67% less likely to use private healthcare facilities, respectively. Similar studies show that well educated mothers are more likely to go to private hospitals seeking for maternal healthcare [18]. The findings point to the power of education in empowering women to seek maternal care and high socioeconomic opportunities both of which reduce the risks of childbirths in areas which lack professional care.

With regard to wealth status, in this study, middle class women were more likely to deliver at home compared to those in the rich wealth quintile households. Women in the low wealth quintile households were less likely to deliver at private healthcare facilities compared to those in the rich wealth quintile households. The findings concur with the report of a cross-country study which used DHS data and showed that the poorest women were over three times more likely to report giving birth at home [24]. Further previous studies have also indicated that poor women rely more on public or governmental health services than on private healthcare facilities [25] compared to women of better living conditions. Private facilities are not affordable to the poor although the quality of services is still questionable [26].

In this study, adequacy of ANC positively correlated with giving birth at private healthcare facilities. Though there are limited sources which examined the effect of adequacy of ANC as a composite indicator, available evidences indicate that the timing of visits and number of visits as well as content of services received have significant effect on mothers' preferences for places to give birth. Studies from Bangladesh [27] and Kenya [13, 28] showed that the less the number of ANC visits is the more likely the woman is to deliver at home. Inadequate services generally prevent women from seeking care during pregnancy or childbirth [29]. Other study findings have indicated that women wishing to give birth in a health facility also make the most use of ANC services [30]. It has been suggested that each antenatal visit creates an opportunity to teach pregnant mothers how to recognize signs of pregnancy complications and how to seek for emergency obstetric care [8].

## 5. Conclusion

Despite the high number of ANC attendances among mothers in the study area, a notable number of pregnant women especially among slum residents still chose to deliver at home. While women's perception of the private sector in Addis Ababa is that it offers better quality services than that offered in the public facilities, still most respondents preferred to deliver in public healthcare institutions despite the general doubts about the quality of services delivered. The preferences were attributed to short distance and perceived as well as experienced low cost of care at public facilities. The observation that utilisation of health facilities was high among younger age groups compared to older women was interesting and factors that demotivate older women from utilising health facilities need to be studied further. To prevent women from reverting back to home delivery, effective communication and particularly counselling of women during ANC visits about the danger signs and complications of pregnancy and childbirth should be enhanced, and concerted effort should be made to encourage every pregnant woman to attend ANC services. Efforts should be directed at the healthcare facilities so that they should provide quality ANC services.

In interpreting this study's findings, it is advisable to consider some of the limitations of the study. The cross-sectional nature of the data does not allow making causal inferences about the relationship between delivery care and the risk factors. It is important to keep in mind that the analysed data includes information reported by mothers only from last pregnancies or childbirths. This study did not collect data about the views and practices of service providers related to quality of services. The study was also limited to the capital city and findings might not reflect the situation of the rest of the country.

## Figures and Tables

**Table 1:** Demographic variables and preferred place of delivery, Addis Ababa, January 2014.

Variables	Place of delivery		
	Home	Public facility	Private facility
*Demographic variables*			
Age group			
15–24	9.6^*∗∗*^	75.1	15.3
25–29	5.8	66.9	27.3
30–49	5.8	67.7	26.5
Number of living children			
0–2	5.1^*∗∗*^	71.7	23.2
3–6	11.7	61.7	26.6
Current marital status			
Currently married	5.5^*∗∗∗*^	65.5	29.0
Cohabiting/living together	6.1	81.7	12.2
Others	15.8	72.6	11.6
Pregnancy intention			
Unintended	13.0^*∗∗∗*^	74.4	12.6
Intended	4.4	67.3	28.2
*Social structure variables*			
Mother's educational status			
No education	19.8^*∗∗∗*^	71.9	8.3
Primary education	7.4	79.5	13.1
Secondary education	2.8	65.5	31.7
Tertiary education	1.5	47.8	50.7
Mother's occupation			
Unemployed	6.9	70.9	22.3
Employed	6.8	64.1	29.1
Wealth index			
Low	6.4	77.9	15.6
Middle	6.7	72.6	20.7
High	7.2	59.3	33.4
Health insurance			
Yes	0.0^*∗∗*^	60.9	39.1
No	4.7	71.6	23.7
Type of residence			
Slum	8.4^*∗∗∗*^	76.3	15.3
Nonslum	3.8	54.5	41.7
Ethnicity			
Amhara	4.8^*∗∗∗*^	68.9	26.3
Guragie	7.1	75.8	17.0
Oromo	8.0	74.1	17.8
Tigraway	1.8	54.5	43.6
Others	15.2	56.5	28.3
Healthcare variables			
Place of ANC visit			
Public facilities	6.7	70.7	22.6
Private facilities	1.7	30.0	68.3
Counselled on danger signs of pregnancy			
Yes	5.1	68.0	26.9
No	7.9	75.3	16.7

*Note*. ^*∗∗*^ *P* < 0.01; ^*∗∗∗*^ *P* < 0.001.

**Table 2:** Odds ratios from multinomial logistic regression for preferences for places to give birth, Addis Ababa, January 2014.

Variables	Preferred place of delivery, AOR [95% CI]	
	Home	Private facility
*Age*	0.90 [0.83, 0.98]^*∗*^	1.02 [0.97, 1.07]
*Number of living children*		
0–2	0.24 [0.10, 0.56]^*∗∗*^	1.20 [0.71, 2.03]
3–6	1	1
*Marital status*		
Currently married	0.70 [0.25, 1.91]	1.33 [0.61, 2.90]
Cohabiting/living together	0.51 [0.16, 1.66]	0.80 [0.32, 2.00]
Others	1	1
*Mother's pregnancy intention *		
Unintended	2.11 [1.03, 4.33]^*∗*^	0.57 [0.34, 0.94]^*∗*^
Intended	1	1
*Mother's educational status*		
No education	3.56 [1.38, 9.21]^*∗∗*^	0.18 [0.08, 0.40]^*∗∗∗*^
Primary education	1.20 [0.51, 2.85]	0.33 [0.22, 0.52]^*∗∗∗*^
Secondary and above	1	1
*Wealth index*		
Low	0.63 [0.29, 1.38]	0.56 [0.37, 0.82]^*∗∗*^
Middle	2.79 [1.18, 6.61]^*∗*^	0.61 [0.31, 1.21]
High	1	1
*Type of residence*		
Slum	1.32 [0.53, 3.24]	0.35 [0.24, 0.51]^*∗∗∗*^
Nonslum	1	1
*Religion*		
Christian	1.19 [0.51, 2.78]	0.42 [0.27, 0.68]^*∗∗∗*^
Muslim	1	1
*High risk pregnancy*		
Yes	0.89 [0.36, 2.19]	0.77 [0.48, 1.23]
No	1	1
*Adequacy of ANC services*		
Inadequate	1.40 [0.31, 6.36]	0.40 [0.24, 0.67]^*∗∗*^
Adequate	1	1

*Note*. (^*∗*^ *P* < 0.05; ^*∗∗*^ *P* < 0.01; ^*∗∗∗*^ *P* < 0.001). ANC: antenatal care, CI: confidence interval, COR: crude odds ratio, and AOR: adjusted odds ratio.
